# Towards Zero Thromboembolic Events After Bariatric Metabolic Surgery

**DOI:** 10.1007/s11695-023-06511-8

**Published:** 2023-03-04

**Authors:** Mohamed Hany, Anwar Ashraf Abouelnasr, Ann Samy Shafiq Agayby, Ahmed Abdelsattar, Bart Torensma

**Affiliations:** 1 Department of Surgery, Medical Research Institute, Alexandria University, Alexandria, Egypt; 2 Department of Bariatric Surgery at Madina Women’s Hospital (IFSO Center of Excellence), Alexandria, Egypt; 3 Al Ahly Hospital, Alexandria, Egypt; 4 Leiden University Medical Center (LUMC), Leiden, The Netherlands

**Keywords:** Thromboembolic, Bariatric metabolic surgery, Deep vein thrombosis, Pulmonary embolism, Porto-mesenteric vascular thrombosis, Low-molecular-weight heparin

## Introduction

Obesity increases the risk of developing venous thromboembolic disease (VTE), including deep vein thrombosis (DVT), pulmonary embolism (PE), and portomesenteric vascular thrombosis (PMVT) [1]. Despite the widespread application of laparoscopy in bariatric metabolic surgery (BMS), VTE is still encountered, with the incidence of symptomatic DVT, PE, and PMVT ranging from 0.2 to 3%, 0.1 to 2%, and 0.3% to 1%, respectively [1, 2]. We provide a brief communication on the current evidence and guidelines derived from our opinion formulated after 15 years of practice and, thereby, postulate novel suggestions for a new regimen.

## Methods

The PubMed and Cochrane databases were searched using the term “*venous thromboembolism AND bariatric surgery AND prophylaxis”*; 225 studies were identified. Of these, 16 were systematic reviews (SR), and 11 SR from 2006 to 2022 were deemed relevant.

When a power calculation of the impact of VTE in clinical research was performed, ((G*power software version 3.1.9.5) (with an alpha of 0.05; beta, 0.8; an incidence, 0.2–2% for VTE)), a minimum of 1566–7846 patients had to be included in a study to assess any form of VTE and treatment effects.

## Results

### Quality of Evidence

The most recent SRs in 2022 was encountered in a study by Zhao et al. They evaluated the outcome of risk of bias and quality of evidence (QoE) for various treatment options, including low-molecular-weight heparin (LMWH) augmented vs. standard dosing, different pharmacologic agents, or extended vs. restricted duration for VTE prophylaxis. In their SR, three were RCTs and 12 were observational studies, with the enrolled sample size varying from 60 to 43,493 (a total of 72,939 patients were enrolled). They revealed that the QoE was low to very low. There was a severe risk of bias, imprecision, and inconsistency. The overall conclusion was that standard LMWH dosing may be effective and safe but that the current evidence is insufficient to support extended prophylaxis [3].

### Cochrane Review

Another SR from Cochrane in 2022 by Amaral et al. was based on a study of the pharmacological interventions for preventing VTE in patients undergoing BMS on regular heparin (higher vs. standard dose), starting before or after surgery, in combination with mechanical treatment, and comparison with another agent, pentasaccharide (fondaparinux), on the outcomes of VTE, bleeding, or all-cause mortality [4].

The studies included in the analyses yielded inconclusive results, given the inherent risk of bias, small sample size, and absence of events. Due to inadequate data, no meaningful conclusions about the hypotheses described above could be made. Hence, the low incidence of VTE and low QoE in the presented studies make it challenging to draw robust conclusions.

### Position Statement of the American Society for Metabolic and Bariatric Surgery

A recent position statement from the American Society for Metabolic and Bariatric Surgery (ASMBS) on perioperative VTE prophylaxis in BMS drew the same conclusion that limited high-quality data were available and that the current practice guidelines suggest some recommendations that are based on available knowledge, peer-reviewed scientific literature, and expert opinion regarding the reasonable use of prophylactic measures for VTE in patients undergoing BMS [5].

## Our Proposed Regimen for VTE Prophylaxis Based on 15 Years of Experience

Due to 15 years of practice in the Medical Research Institute, Alexandria University, and Madina Women’s Hospital, Alexandria, Egypt, we noted continuous modifications and changes in doses and duration of heparin, which were investigated. The investigation was guided by the occurrence of events, culminating in the following conclusions and postulated regimen.

### Baseline Characteristics

Our clinic had extensive experience, with 11,434 patients undergoing surgery before beginning the new regimen. Of these, 48.2% underwent laparoscopic sleeve gastrectomy (LSG), 29.1% had Roux-en-Y gastric bypass (RYGB) (8.6% underwent one-anastomosis gastric bypass (OAGB), and 14.1% had revision surgeries. Patients with VTEs had a BMI ranging from 45 to 56 kg/m^2^. The events occurred from days 9 to 24 postoperatively.

### Incidence of VTE

The incidence of VTE before the start of the new regimen was 0.14%. Of this, 0.11% were reportedly mesenteric vascular occlusions (MVO), and 0.03% were PE. All diagnoses occurred between 9 and 16 and 11 and 24 days postoperatively. The diagnosis was made using CT pulmonary angiography (CTPA) and CT of the abdomen with mesenteric angiography. A complete workup of the signs and symptoms of the PTE diagnostic criteria is presented in Appendix 1.

In total, two mortalities (0.02%) were recorded due to MVO. From December 2020 to November 2022, a total of 2535 patients underwent surgery at our center, and they had zero (0.0%) clinical thromboembolic events.

## The Regimen for VTE Prophylaxis and Risk Groups

We categorized the patients into two groups:

- Group one (high risk): patients with obesity + BMS with no additional risk factors*.
- Group two (very high risk): patients with obesity + BMS + additional risk factors* (*risk factors include age > *60 years; current smoking; a history of VTE, thrombophilia, chronic venous insufficiency, varicose veins, diabetes, congestive heart failure, COPD, immobility, cancer, chemotherapy, inflammatory diseases, estrogen and hormonal medications, and fractures; and congenital hypercoagulable state).*

All patients undergoing BMS were considered to be at high risk (and not at average or moderate risk) for VTE at all times. Of the patients operated upon, 44.7% were in group two, indicating the gravity of the problem in our clinic.

After discharge, a special “postdischarge team” follows up on all the operated patients daily by phone for ten days straight on all topics related to the procedure. This involves asking several questions, including the patient’s temperature, fluid intake, and use of the anticoagulation medication.

## Rationale for the VTE Prophylaxis

In short, our summarized rationales, whereby more background information is available to us.

### LMWH and Dosing

Our clinic used LMWH because it was more effective than unfractionated heparin in preventing post-BMS VTE [6]. Evidence for more benefit in higher dosages of LMWH was used. For example, therapeutic levels of anti-factor Xa have been achieved with enoxaparin 60 mg compared to 40 mg per day [7]. A lower rate of DVT has been reported with enoxaparin 40 mg twice daily than with 30 mg twice daily [8]. Therefore, our clinic preferred a starting dosage 30% higher than the standard starting dosage owing to the high-risk profiles of our patients [9].

### Total Body Weight

Corresponding to the patient’s profiles, total body weight-based (TBW) dosing was applied rather than BMI-based dosing to achieve better tailoring and personalization.

### Duration

As a standard duration, a complete course lasting 21–28 days postoperatively is deemed desirable (Table 1).

**Table 1:** Presentation of the suggested new regimen and old regimen for LMWH (enoxaparin) post-bariatric metabolic surgery VTE prophylaxis

Total body weight (TBW) in kg New regimen	Group one (high risk)	Group two (very high risk)
Up to 100 kg	60 mg once/day for 21 days	80 mg once/day for 14 days followed by 60 mg once/day for 14 days
	**(21 days total)**	**(28 days total)**
101−119 kg	80 mg once/day (or 40 mg/12 hrs) for 21 days	100 mg/day (60 mg am + 40 mg pm) for 14 days followed by 80 mg once/day for 14 days
	**(21 days total)**	**(28 days total)**
120−139 kg	100 mg/day (60 mg am- 40 mg pm) for 21 days	60 mg/12 hrs for 14 days followed by 80 mg once/day for 14 days
	**(21 days total)**	**(28 days total)**
140−159 kg	60 mg/12 hrs for 21 days	80 mg/12 hrs for 14 days followed by 80 mg once/day for 14 days
	**(21 days total)**	**(28 days total)**
160−200 kg	80 mg/12 hrs for 14 days followed by 80 mg once/day for 14 days	100 mg/12 hrs for 14 days followed by 60 mg/12 hrs for 14 days
	**(28 days total)**	**(28 days total)**
More than 200 kg	80 mg/12 hrs for 14 days followed by 60 mg/12 hrs for 14 days	100 mg/12 hrs for 14 days followed by 80 mg/12 hrs for 14 days
	**(28 days total)**	**(28 days total)**
Note to the new regimen if there is an allergy to enoxaparin, we shift to fondaparinux as follows: • Group 1 for 21 days: - BMI < 50 kg/m^2^ daily dose of 2.5 mg - BMI >50 kg/m^2^ daily dose of 5 mg • Group 2 for 28 days - BMI < 40 kg/m^2^ daily dose of 2.5 mg - BMI >40 kg/m^2^ daily dose of 5 mg		
Old regimen (enoxaparin)		
BMI Old regimen (enoxaparin)	Dose	
BMI 30-39kg/m2	Standard prophylactic dose, i.e., 30mg/12hrs or 40 mg once OR weight-based dosage, i.e., 0.5 mg once or twice according to the level of VTE risk	
BMI >40	Empirically increase the standard dose by 30%, i.e., from 30mg/12 hours to 40 mg/12 hours OR weight-based dosage, i.e., 0.5 mg once or twice according to the level of VTE risk	
High VTE-risk bariatric surgery with BMI < 50	40 mg/12 hours	
High VTE-risk bariatric surgery with BMI > 50	60 mg/12 hours	

## Preoperative VTE Prophylaxis

Twelve hours prior to BMS, the patient received 40 mg of enoxaparin subcutaneously, and this was continued for 12 h postsurgery.

### Oral Anticoagulant Presurgery

In case the patients had received oral anticoagulant before surgery, a rapid-acting short half-life anticoagulant (enoxaparin) was also administered to minimize the risk of bleeding. As the half-life of the anticoagulant effects of warfarin is 36–42 h, it would take at least 5 days for the anticoagulant effect of warfarin not to be active any longer after discontinuation.

Additionally, enoxaparin should be started 36–48 h after the last warfarin dose.

To complete bridging, a therapeutic dose of 60 or 80 mg twice daily was required, with the last dose administered 12 h before surgery. Prophylactic doses were used when the risk of discontinuation of an anticoagulant was low in the context of cardioembolic sequelae.

### Regimen on Direct Oral Anticoagulants (DOACs)

DOACs (e.g., apixaban, dabigatran, edoxaban, and rivaroxaban) possess a rapid onset of action of approximately 1–3 h to peak concentration and a shorter half-life than warfarin.

In line with the general prescription, the DOAC was stopped 48 h before the BMS. In patients at risk of atrial fibrillation, those with a history of DVT, and those with prosthetic heart valves, therapeutic cover with a dose of enoxaparin 40 mg was initiated. The last dose was administered 12 h before BMS.

### Other drugs

Aspirin was not stopped before BMS, and clopidogrel was stopped 5 days before surgery and bridged with a prophylactic dose of enoxaparin.

## Special Circumstances

Extensive monitoring was necessary for the following elements:

1. Elastic compression class 1, knee-high type (15–18 mmHg), was measured and used in all cases for DVT prevention (except in cases of lymphedema) with a recommendation of early ambulation.
2. The drug dosage during the menstrual period was kept constant, corresponding to the weight and risk of the patients unless profuse bleeding was encountered. The doses, when patients had increased menstrual bleeding, were lowered by 20 mg. Its administration was changed to once instead of twice daily to maintain the near-average amount prescribed to the patient. At the end of the menstrual period, the standard prescribed dose prior to menstruation was recommenced.
3. In patients with renal conditions, the regimen was reduced to half of the doses mentioned in Table 1 [10].
4. If melena or hematemesis occurred during administration or hospital stay, the drugs were discontinued and resumed 2 days poststabilization. The entire therapeutic background for the special circumstances is presented in Appendix 2.

The dosing and duration of the new and old regimens of LMWH (enoxaparin) post-BMS for VTE prophylaxis are described in Table 1.

## Postoperative Bleeding

The incidence of postoperative bleeding during admission was in the old regimen 0.22% (26 out of 11,434 patients), whereby in the new regimen, this was 0.15% (4 out of 2535 patients).

The incidence of bleeding after discharge while on anticoagulants was in the old regimen, 0.008% (1 patient), and in the new regimen, 0.11% (3 patients). In the old regimen, the patient had undergone LSG and experienced hematemesis on postoperative day 4 but remained stable. The patient was readmitted and followed up for 2 days before being discharged.

The three patients in the new regimen had undergone OAGB and presented on postoperative days 5–8 with melena and decreased hemoglobin levels. Re-operation or exploration was not necessary, and the hemodynamics of the patients were stable. They were readmitted to the hospital, received one packed RBC transfusion, and were discharged.

To outweigh what has more impact on the patient’s well-being, studies showed that approximately 20% of the counted thromboembolic events are considered fatal, with 40% leading to severe complications like permanent disability [11].

A systematic review by Rocha et al. evaluated 36 studies; in 8 studies, a fatal incidence was described after VTE. The fatality rate was between 25 and 58%, calculated from the incidence of VTE in the described cases [12]. In contrast, only about 3% of major postoperative bleeding events are known to be fatal, with most patients reporting full or uneventful recovery after hemorrhagic complications [3, 11]. Therefore, the risk of procedure-related VTEs is thought to outweigh the risk of bleeding events during the regimen.

## Conclusion

Adopting the local practice in our clinic is advocated within the guidelines suggested by the ASMBS. Furthermore, every institution can implement the principles based on the prevalent clinical indications [8]. Here, the general objective was to create guidelines to help lower the incidence of VTE. The BMS community should consider commencing a worldwide collaboration to enable sufficient study power and learn from the lessons to create higher QoE in RoB assessments. Such an international liaison may be the only way to provide a new guideline for VTE prophylaxis, whereby sharing data with centers would be crucial to spread the knowledge and impact of the studies.

## Data Availability

Data is available on request by the corresponding author.

## Appendix 1: Signs and Symptoms as PTE Diagnostic Criteria

During follow-up, the outpatient treatment team paid extra attention in the early postoperative period to detect and manage complications; accordingly, all complications were reported to the clinic and discussed with the multidisciplinary team.

Therefore, the diagnosis of thromboembolic events, including pulmonary embolism (PE) and mesenteric vascular occlusion, was cautiously monitored as an essential benchmark outcome in our clinic.

Symptoms described by PE patients were as follows: shortness of breath (3 patients), back pain (1 patient), excessive sweating (3 patients), chest pain (2 patients), palpitations (3 patients), fever (2 patients), syncope (1 patient), and lightheadedness (1 patient).

Signs of PE were as follows: tachycardia, tachypnea, hypotension (1 patient), cyanosis (1 patient), fever (1 patient), calf pain (1 patient), widespread polyphonic wheeze (2 patients), and a loud 2nd heart sound (S2) (1 patient).

For patients who were admitted to ICU, the following findings were observed:

- Pulse oximetry showed hypoxemia
- Chest X-rays revealed non-specific atelectasis
- Blood gas testing indicated hypocapnia
- ECG showed tachycardia and various ST-T wave abnormalities
- Elevated D-dimer levels were noted
- CT pulmonary angiography was diagnostic in cases showing filling defects in the segmental and lobar vessels
- Duplex ultrasonography for the legs was performed and was negative
- Echocardiography was performed, and cardiac markers were also tested

Furthermore, the diagnosis depends on how a patient presents their signs and symptoms; this could lead to a high index of suspicion, but the diagnosis is always confirmed by CTPA.

### MVO

MVO was diagnosed in 13 patients. Two patients displayed total occlusion of the portal vein (PV) main trunk, its branches, the superior mesenteric vein (SMV), and the tributaries of the SMV.

The remaining patients suffered partial PV and partial SMV-splenic vein thrombosis. Mortality was documented in two patients with small bowel gangrene. Of all the patients, 92% presented with abdominal pain, 46% with nausea, 25% with vomiting (two turbid bloody vomits), 29% with diarrhea, and 11% with rectal bleeding. Approximately one-third of the patients reported abdominal pain, fever, and hemoccult-positive stools. Presenting with signs included tachycardia, fever, and diffuse abdominal tenderness. Blood tests demonstrated leukocytosis and elevated C-reactive protein (CRP) levels. The diagnosis was confirmed with an abdominal CT with oral and IV contrast.

Comprehensive biphasic CT angiography includes the following essential signs:

1. Precontrast scans revealed vascular calcification, hyperattenuating intravascular thrombus, and intramural hemorrhage.
2. The arterial and venous phases demonstrate thrombus in the mesenteric arteries and veins, abnormal bowel wall enhancement, and embolism or infarction of other organs.
3. Multi-planar reconstructions (MPR) for assessing the origin of the mesenteric arteries.

The findings from the CT angiography were as follows:

- Total occlusion of PV and SMV (two cases)
- Partial PV occlusion and SMV (six cases)
- SMV thrombosis (three patients)
- SMV and splenic vein occlusion (one patient)
- Free intraperitoneal fluid (mild to moderate)—all patients.
- Pleural effusion, bilateral (six cases)
- Intestinal dilatation and thickness (segmental)
- Pneumatosis intestinalis and portal venous gas in two cases of total PV occlusion and SMV occlusion.

Two mortalities were recorded, and the rest were therapeutically managed with anticoagulants. Three laparotomies were performed with segmental resection of the small bowel wall in two patients. One patient underwent exploration, but total gangrene of the entire small bowel was noted, and no further management was initiated.

## Appendix 2: Entire Therapeutic Background for Special Circumstances

1. Elastic compression class 1, knee-high type (15–18 mmHg), was used in all cases (except in cases with lymphedema, significant limb deformity, unusual leg size or shape preventing correct fit, severe leg edema, dermatitis, suspected or proven peripheral arterial disease, and peripheral neuropathy), whereby all the patients were recommended early mobilization.
2. The drug dosage during the menstrual period was kept the same, corresponding to the weight and risk of the patient unless the bleeding was profuse. In such cases, the doses when patients had increased menstrual bleeding were lowered by 20 mg, with administration adjusted to once instead of twice a day to maintain the standard dose prescribed to the patient. At the end of the menstrual period, the standard prescribed dose as before the beginning of menstruation was recommenced.
3. For patients diagnosed with renal impairment, the regimen was reduced to half the doses mentioned in Table 1. Creatinine clearance (CrCl) was measured in these patients, and the dose was adjusted according to a 30 mL/min cutoff value. An FDA-approved two-tiered dosing strategy with a lower dosage is recommended for patients with a CrCl ≤ 30 mL/min.
4. If melena or hematemesis occurred during administration or hospital stay, the drugs were discontinued and resumed after stabilization for 2 days once the complete blood count (CBC) results were within the normal range.
5. When postoperative bleeding was encountered, a blood transfusion was performed, and the dosage based on described regimen was continued poststabilization.

Indications such as tachycardia and decreased hemoglobin level must be further considered, and the cause of bleeding must be conventionally controlled by re-laparoscopy. The initiation of conservative therapy with transfusion, CBC, and IV vitamin K monitoring, and tranexamic acid administration was done before the transfusion.
